# Major Constituents of Cannabis Vape Oil Liquid, Vapor and Aerosol in California Vape Oil Cartridge Samples

**DOI:** 10.3389/fchem.2021.694905

**Published:** 2021-06-21

**Authors:** Weihong Guo, Gordon Vrdoljak, Ven-Chi Liao, Bahman Moezzi

**Affiliations:** Food and Drug Laboratory Branch, California Department of Public Health, Richmond, CA, United States

**Keywords:** vape oil, EVALI (e-cigarette or vaping product use-associated lung injury), aerosol, GC-MS, vapor, nontarget, toxin, delta-9 tetrahydrocannabinol

## Abstract

During the E-cigarette or Vaping product use Associated Lung Injury (EVALI) outbreak of August 2019 to February 2020, the California Department of Public Health, Food and Drug Laboratory Branch received numerous cannabis vape oil cartridge investigation samples from throughout the state. Many of these products were directly linked to patients; others were collected as part of investigations. We determined the major ingredients and additives in twelve unused cannabis vape oil cartridge samples obtained before (*n* = 2) and during the EVALI outbreak (*n* = 10) in California from September 2018 to December 2019. We tested for major constituents in vape oil liquid, vape oil vapor, and vape oil aerosol phases. A nontargeted Gas Chromatography Mass Spectrometry direct injection screening method was developed for vape oils, a headspace heating module used for vape oil vapors and a solid-phase microextraction (SPME) vaping rig for aerosols generated by vaping. We have identified more than 100 terpenes and natural extracts, 19 cannabinoids, and other potential toxic additives such as Vitamin E Acetate, Polyethylene Glycols, and Medium Chain Triglycerides. We determined more terpenes and minor cannabinoids can be produced *via* vaporizing and aerosolizing the vape oil. Delta9-THC and potential toxic additives were found at lower levels in the vapor and aerosol than in the vape liquid.

## Introduction

Medical cannabis became legal in California since 1996 under Proposition 215–the Compassionate Use Act (CUA). In November 2016, 57% of voters passed Proposition 64–the Adult Use of Marijuana Act (AUMA), leading to recreational cannabis sales in California (CDPH Legislation). Since then, cannabis products are expanding into many innovative forms consumed by both medical patients and recreational cannabis users. A variety of cannabis products are available in California including joints, beverages (in different flavors), concentrates/distillate, vape cartridges (in different flavors), topicals, oral supplements, tinctures, capsules, and various infused edibles such as candies/chocolates, mint/chews, dried meat, crackers, dairy product and baked goods. Among these cannabis products, vape oil cartridges are particularly popular as they share the electronic nicotine delivery systems (ENDS). This method of consumption and delivery of tetrahydrocannabinol (THC) is claimed to be safer and more efficient than other products. However, ENDS use is not without short or long-term adverse effects due to additional chemicals generated in the system and the strength of active contents (Rehan et al., 2018; Livingston et al., 2019).

ENDS were first invented by Lik Hon in Hong Kong in 2003 and was entered in Europe and the United States in 2006 (PRLOG, Hon Lik, 2010). It became popular in 2012 after tobacco manufacturers joined the market (Hajek et al., 2014). There are many terms used to describe ENDS such as vapes, vaporizers, vape pens, hookah pens, electronic cigarettes, etc. (USFDA, 2020a). It consists of an atomizer as the heating element, a wick, a battery power source, and a cartridge or tank container. Instead of containing nicotine, cannabis vape cartridges typically contain a mixture of cannabinoids, terpenes, various solvents used as thinning agents, and flavoring additives. By pressing the power button, the vape oil is heated to create an aerosol that the user inhales. Overall, e-liquid aerosol contains fewer types and lower levels of toxicants than smoke from combustible tobacco cigarettes (Hajek et al., 2014; The National Academies of Sciences, E et al., 2018). However, the recent outbreak of EVALI has triggered health concerns in the vaping community.

The EVALI outbreak was first identified in August 2019 and peaked in September 2019 followed by a gradual, but persistent decline (CDC Update, 2019; Heinzerling et al., 2020). As of February 18, 2020, a total of 2,807 hospitalized EVALI cases or deaths have been reported to CDC from all 50 states. National and state data from patient reports show THC containing e-cigarette or vaping products, particularly from informal sources, online dealers, and illicit market are linked to most EVALI cases. Vitamin E acetate has been found in these product samples tested by FDA and state laboratories. It was also found in patient lung fluid samples collected from various states and tested by CDC (Heinzerling et al., 2020; Blount et al., 2020; Duffy et al., 2020). The surge of the EVALI outbreak strongly shows the need of routine investigation of cannabis products on the markets and in-depth research for the safe use of e-cigarettes or vaping products.

Numerous studies have been conducted on e-liquids containing nicotine using propylene glycol (PG) and Vegetable Glycerin (VG) as solvent thinning agents (also called cutting agents for the ease of vaporizing) with added flavonoids (The National Academies of Sciences, E, 2017; The National Academies of Sciences, E et al., 2018; LeBouf et al., 2018; Strongin, 2019). These studies revealed the concerns of Harmful and Potentially Harmful Constituents (HPHCs) formation during heating and aerosolization of the e-liquids. Such studies cannot be directly applied to cannabis products as they have different major constituents such as THC (50–80% concentration) and terpenes. From limited studies on cannabis vape cartridges, ketene as an exceptionally toxic gas may be a potential byproduct in the aerosol of vape cartridges containing Vitamin E acetate (Attfield et al., 2020; Strongin, 2020; Wu and O’Shea, 2020). Poklis and Peace et al. also found synthetic cannabinoids in vape liquids (Peace et al., 2017; Poklis et al., 2019). In addition, residual solvents, pesticides, heavy metals and other toxic chemicals can be concentrated during the cannabis extraction process and remain in the vape oil (Raber et al., 2015; Cannabis.net., 2020). Many vape pens have poor temperature control and the vape cartridge content may be heated to beyond the optimum temperatures, or even to the point of combustion (Wagner et al., 2020). Consequentially, users may inhale cannabis smoke containing carbon-monoxide, tar, ammonia, heavy metals and other by-products that are harmful to the lungs and respiratory health (King, 2020). Therefore, to expand the understanding and collect more knowledge to ensure product safety for consumers, the National Academies of Sciences research group suggested studies focus on cannabis products containing cannabis, cannabinoids, or THC (The National Academies of Sciences, E, 2017).

In the current study, we investigated twelve cannabis cartridge samples obtained from various dispensaries in California from September 2018 to December 2019. Among these twelve samples, two were prior to the EVALI outbreak and ten were during the EVALI outbreak. We analyzed the composition of the vape oils focusing on volatile and semivolatile chemicals. By using a nontargeted gas chromatography mass spectrometry (GC-MS) screen method, we were able to detect and identify unknown and suspicious compounds in addition to cannabinoids, terpenes and other known major additives. We hypothesized there were different constituents in the cartridges collected before and during the EVALI outbreak. As manufacturers drive to improve profits, they may alter product formulations by using cheaper ingredients in their products, and these new ingredients may pose health risks to consumers. The new ingredients should have a safety assessment by following U.S. Department of Health and Human Services, Food and Drug Administration (USFDA) guidance prior adding them into the products [USFDA, 2020b. Nonclinical, 2020b]. A nonclinical toxicity assessment can also help to address the potential toxicity of chemicals, especially novel chemicals and impurities generated from heating in product delivery systems.

In this study, we also tested vape oil composition in its vapor and aerosol phases using headspace heating and solid-phase microextraction (SPME) GC-MS analyses. We hypothesized that there were some differences among original vape oil liquid, vapor and aerosol. By heating and aerosolizing the vape oil, we simulated the battery powered vape pen conditions used by consumers. This may help to determine the major constituents and their amount in vapor or aerosol that get into user’s lungs. To the best of our knowledge, this is the first study that compared the major constituents in unused vape oil to those of its vaporized and aerosolized forms.

## Method and Materials

### Cannabis Vape Cartridge Samples

In this study, we investigated twelve cannabis vape cartridges obtained through the California state surveillance program from September 2018 to December 2019. Sample details are listed in Table 1. Each of the twelve cannabis vape cartridges went through analyses in its oil liquid, vapor, and aerosol phases for the determination of the major constituents.

**TABLE 1:** New and unused vape cartridge samples obtained from September 2018 to December 2019 in California dispensaries.

Sample ID	Weight (g)	Condition	Labeled THC/CBD	Flavor name	Collection Date	Origin
F18CTS035	1.0	Full, high viscosity	N/A	Sour diesel	September 2018	San Diego
F18CTS046	0.5	Full, high viscosity	THC 86.6%	Lemon tree	December 2018	Oakland, CA
F1909018-005	1.1	Full, medium viscosity	THC 92.23%	Purple punch	September 2019	Lake Forest, CA
F1909018-008	1.1	Full, low viscosity	THC 90.99%	Lemon berry	September 2019	Los Angeles, CA
F1910011-001	0.5	Half full, dryness	N/A	Jack herer	October 2019	Rancho Cordova, CA
F1910013-001	0.5	Half full, high viscosity	N/A	Citron OG	October 2019	Rancho Cordova, CA
F1912005-002	1.0	Full, high viscosity	N/A	Blue dream	December 2019	Los Angeles, CA
F1912005-003	0.5	Half full, high viscosity	THC 65.21%, CBD 0.67%	Pure organics sativa	December 2019	Los Angeles, CA
F1912005-004	1.0	Full, high viscosity	N/A	Tangie sativa	December 2019	Los Angeles, CA
F1912012-001	1.0	Full, dryness	THC 84.35%, CBD 0.23%	Purple punch indica	December 2019	Los Angeles, CA
F1912011-001	0.5	Full, low viscosity	N/A	Gorilla glue	December 2019	Los Angeles, CA
F1912013-004	0.5	Half full, high viscosity	THC 80–85%	Topanga canyon OG	December 2019	Los Angeles, CA

### Nontargeted GC-MS Screen for Cannabis Cartridge Vape Oil

The nontargeted GC-MS screen method uses a full scan mode in MS to tentatively identify known and unknown/nontargeted chemical substances in a sample based on a match to an established mass spectral library. This method has been used for toxin screen in United States Department of Agriculture Food Safety and was validated in our laboratory for cannabis vape oil samples with modifications (United States Department of Agriculture Food Safety and Inspection Service, 2013). In general, minimum of 0.5 g of cannabis vape oil was taken out from cartridge device by centrifugation to a 15 ml tube. Approximately 10 mg of sample was aliquoted and accurately weighed into a 1.5 ml Eppendorf vial after homogenization by gently stirring with a pipette tip. Aliquoted samples were diluted in methanol by one thousand times (1000 x) and spiked with an internal standard mix (Triphenylphosphate and Phenanthrene-d_10_, Sigma-Aldrich), before being injected on Agilent GC7890B coupled with MS5977B (Agilent, Santa Clara, CA). Quality control samples containing toxin standards (Nicotine, Parathion, Codeine, and Strychnine, from Sigma-Aldrich and AccuStandard) were included in each sample batch.

An injection of 1 µl of each sample was injected on the GC injection port using splitless mode. Chromatographic separation was achieved in a 30 min run time using a DB-5MS column (30 m × 0.25 mm × 0.25 µm, Agilent) with 1 ml/min helium flow. The oven temperature program was set at 60^o^C at 1 min, followed by a 12°C/min ramp to 320°C and hold for 7.3 min. The transfer line temperature was set at 280°C, the ion source temperature at 250°C, and EI ionization energy at 70 eV. Mass spectral data was acquired in the scan mode from 25 to 550 m/z at a speed of 2.8 scan/s. Tentative compound identifications are based on a comparison of electron impact mass spectra with the Wiley11/NIST 2017 Mass Spectral libraries and Cayman Spectral library. The match criteria from the compound mass spectra to the database must have a fit of greater than 90% match ratio and visually verified by the analyst. All major cannabinoids identified by the library match were confirmed using the cannabinoid standards purchased from Cerillant and Cayman Chemical. Nineteen major terpenes available in cannabis terpene mix and additives such as VEA, MCTs, PEGs identified in samples were confirmed with the standards purchased from Sigma-Aldrich, and Emerald Scientific.

### Headspace GC-MS Screen for Cannabis Vape Oil Vapor

The headspace GC-MS method uses a headspace PAL3 autosampler with a heating agitator to simulate the heating effects of vaping on vape oils and introduces the sample vapor to a GC-MS (Agilent 7890B/5977B). According to Chen et al., the heating temperatures were influenced by power settings, coil wetness conditions (the fullness or amount of vape oil in the cartridge), and nicotine e-liquid compositions (Chen et al., 2018). Propylene Glycol (PG) is one of the major solvents in e-liquids (Prochaska, 2019). Under the test conditions using a PG e-liquid, coil temperatures ranged from 322 to 1008°C for dry cartridge conditions, 145–334°C for wet-through-wick conditions, and 110–185°C for full-wet conditions (Chen et al., 2018). Based on the fullness of tested vape cartridges in the current study and user’s practical consumption scenario, the wet-through-wick conditions are the most common. In addition, most terpenes and cannabinoids boiling points are from 150–200°C and around 200°C has the most desirable medical effects for users (Post, 2020). Therefore, the temperature of 200°C was set on the heating agitator to generate cannabis oil vapor.

Approximately 10 mg of sample was accurately weighed into a 20 ml headspace vial after homogenization by gently stirring with a pipette tip. Aliquoted samples were spiked with an internal standard mix (Triphenylphosphate and Phenanthrene-d10, Sigma-Aldrich). Quality control samples were included in each sample batch run. Each sample was heated at 200°C in the agitator block for 15 min and 1 ml of vapor was injected into the GC-MS for analysis using the same GC-MS screen method for cannabis vape oil.

### SPME GC-MS Screen for Cannabis Vape Oil Aerosol

SPME is an innovative, solvent-free sample preparation technology that uses a coated fiber to extract volatiles and non-volatiles from different sample matrices. During the process, the SPME fiber concentrates the analytes from the sample to the fiber. By injecting the SPME fiber directly into the GC port, the analytes on the fiber are thermally desorbed in the GC injector and then rapidly flushed to the GC column (Sigma, 2020). This technique was used to collect the analytes in vape oil aerosols.

To determine the chemical compounds inhaled by the consumers when vaping cannabis oils using battery powered vape pens, a common vape pen was purchased (Brillian) which has three different voltage settings (3.7, 3.9 and 4.2 V) and the 3.9 V was used for all the samples. Nicotine cartridge samples were used as the quality control samples throughout the SPME GC-MS analysis.

An e-liquid aerosol trap system was set up according to Peace et al. (2016) and Peace et al. (2018), with modifications. In brief, two Erlenmeyer flasks were connected in tandem to a vacuum with an air flow rate of 1457 ml/min. Deionized water was added to each trap flask and a gas dispersion tube bubbles the aerosol into the water. Glass wool was placed in between the two traps to contain the aerosol in the first trap. A 100 µm polydimethylsiloxane (PDME) coated SPME fiber injector (fused silica fiber core in red hub, Supelco) was inserted through a septum in the first trap to absorb the aerosol cloud. The fiber inside the injector was exposed into the trap while the vape cartridge is activated by the battery power and the aerosol fills the trap. The SPME fiber was held in the trap for about 2 min while the vape pen is activated for 5 times (5 puffs). The fiber was retracted after the aerosol clouds disperse from the last puff. It was then manually inserted into the injection port with a 15 min thermal desorption time on a GC-MS (Agilent 7890B/5977B) and analyzed using the same GC-MS screen method for cannabis vape oil.

## Results and Discussion

### Nontargeted GC-MS Screen for Cannabis Cartridge Vape Oil

Twelve vape oil liquid samples were tested for constituents. Terpenes, terpenoids, flavor and fragrance agents, cannabinoids, and many other additives were detected (Tables 2 and 3). We observed that the flavor names and cannabinoids listed on their respective package did not necessarily correspond with the terpene types and cannabinoids found in samples. For example, product type Blue Dream is high in myrcene, known for its relaxing and sedative effects and Sour Diesel is high in both myrcene and limonene, a combination known for its energizing and stress-relieving effects (Erickson, 2019), but we did not find the stated terpene types in those samples. Therefore, the package descriptions may merely serve as a marketing tool to attract consumers who seek for those added benefits.

**TABLE 2:** Terpenes detected in vape liquid, vapor and aerosol samples.

Compound names	CAS#	Formula	MW	Functions	Liquid^a^	Vapor^a^	Aerosol^a^
Caryophyllene	000087-44-5	C15H24	204.4	Terpene	12	12	10
Alpha-Bisabolol	000515-69-5	C15H26O	222.4	Terpene	11	11	8
Linalool	000078-70-6	C10H18O	154.3	Terpene	10	10	9
Alpha-Humulene/Humulene	006753-98-6	C15H24	204.4	Terpene/Flavor	9	12	10
D-limonene	005989-27-5	C10H16	136.2	Terpene/Flavor	8	9	10
Phytol	000150-86-7	C20H40O	296.5	Terpene	8	9	x
Caryophyllene oxide	001139-30-6	C15H24O	220.4	Terpene	8	7	6
Eudesma-3,7(11)-diene/Selina-3,7(11)-diene	006813-21-4	C15H24	204.4	Terpene	6	11	10
Fenchol	001632-73-1	C10H18O	154.3	Terpene	6	7	9
Nerolidol/(+)-Nerolidol	007212-44-4/000142-50-7	C15H26O	222.4	Terpene/Flavor/Fragrance	6	7	3
Squalene	000111-02-4	C30H50	410.7	Natural extract	6	4	1
Gamma-Selinene	000515-17-3	C15H24	204.4	Terpene	5	7	5
Beta-Myrcene	000123-35-3	C10H16	136.2	Terpene	5	5	8
Terpinolene	000586-62-9	C10H16	136.2	Terpene/Flavor/Fragrance	4	6	10
Alpha-Terpineol/(+)-Alpha-Terpineol	000098-55-5/007785-53-7	C10H18O	154.3	Terpene/Flavor/Fragrance	4	2	1
2(10)-Pinene/Beta-Pinene	000127-91-3	C10H16	136.2	Terpene	3	8	9
Alpha-Selinene/(+)-Alpha-Selinene	000473-13-2	C15H24	204.4	Terpene	3	5	5
Alpha-Eudesmol	000473-16-5	C15H26O	222.4	Terpene	3	4	3
Guaiol	000489-86-1	C15H26O	222.4	Terpene	3	4	3
Valencene	004630-07-3	C15H24	204.4	Flavor/Fragrance	3	4	3
Beta-Maaliene	000489-29-2	C15H24	204.4	Terpene	3	**2**	2
Copaene	003856-25-5	C15H24	204.4	Terpene	2	7	8
Borneol/Endo-borneol	000507-70-0	C10H18O	154.3	Terpene	2	5	3
Neophytadiene	000504-96-1	C20H38	278.5	Terpene	2	5	1
Bulnesol	022451-73-6	C15H26O	222.4	Terpene	2	4	3
m-Camphorene	020016-73-3	C20H32	272.5	Terpene	2	3	x
Delta-Guaiene/Alpha-Bulnesene	003691-11-0	C15H24	204.4	Terpene	2	2	5
Gamma-Eudesmol	001209-71-8	C15H26O	222.4	Terpene	2	2	x
Junipercamphor	000473-04-1	C15H26O	222.4	Terpene	2	2	1
Alloaromadendrene	025246-27-9	C15H24	204.4	Terpene	2	1	2
Beta-Cadinene/(-)-beta-Cadinene	003858-53-5/523-47-7	C15H24	204.4	Terpene/Flavor/Fragrance	2	1	x
Isoledene	095910-36-4	C15H24	204.4	Terpene	2	x	1
2-Pinene	000080-56-8	C10H16	136.2	Terpene	1	9	9
3-Carene/Delta-3-carene	013466-78-9	C10H16	136.2	Terpene/Flavor/Fragrance	1	3	4
Isoborneol	000124-76-5	C10H18O	154.3	Terpene	1	3	3
(E)-Beta-Famesene	018794-84-8	C15H24	204.4	Flavor/Fragrance	1	2	2
Aromandendrene	000489-39-4	C15H24	204.4	Terpene	1	2	2
Butylated hydroxytoluene	000128-37-0	C15H24O	220.4	Precervative	1	2	2
Citronellol	000106-22-9	C10H20O	156.3	Flavor/Fragrance	1	2	1
Eudesma-4(14),11-diene	058893-88-2/17066-67-0	C15H24	204.4	Terpene	1	2	3
Farnesol	004602-84-0	C15H26O	222.4	Terpene/Flavor/Fragrance/Cosmetic	1	2	x
p-Camphorene	020016-72-2/000532-87-6	C20H32	272.5	Terpene	1	2	x
l-Menthone	000089-80-5	C10H18O	154.3	Flavor/Fragrance	1	1	1
(E,Z)-Alloocimene	007216-56-0	C10H16	136.2	Flavor/Fragrance	1	1	3
Alpha-Cubebene/(-)-Alpha-Cubebene	017699-14-8	C15H24	204.4	Terpene	1	1	3
Alpha-Gurjunene	000489-40-7	C15H24	204.4	Fragrance	1	1	3
Benzyl alcohol	000100-51-6	C7H8O	108.1	Flavor enhancer/Precervative	1	1	x
Beta-Panasinsene	997220-91-1/56684-97-0	C15H24	204.4	Terpene	1	1	1
D-Carvone	002244-16-8	C10H14O	150.2	Terpene/Flavor/Fragrance	1	1	1
Trans-Alpha-Bergamotene	013474-59-4	C15H24	204.4	Terpene	1	1	1
Beta-Amyrin	000559-70-6	C30H50O	426.7	Terpene	1	x	x
Calamenene/Cis-Calamenene	072937-55-4	C15H22	202.3	Terpene	1	x	1
Cetene	000629-73-2	C16H32	224.4	Terpene	1	x	x
Gamma-Cadinene/Gamma-Muurolene	030021-74-0	C15H24	204.4	Terpene	1	x	3
Methyl salicylate	000119-36-8	C8H8O3	152.1	Terpene/Flavor/Fragrance	1	x	x
Trans-Verbenol	001820-09-3	C10H16O	152.2	Flavor/Fragrance	1	x	x
Triacetin	000102-76-1	C9H14O6	218.2	Flavor/Biocide	1	x	x
Viridiflorene	021747-46-6	C15H24	204.4	Terpene	1	x	x
3-Methylcyclopentyl acetate	024070-70-0/035897-13-3	C8H14O2	142.2	Terpene	x	6	x
Epi-γ-Eudesmol	117066-77-0	C15H26O	222.4	Terpene	x	6	2
Piperitenone	000491-09-8	C10H14O	150.2	Terpene/Flavor/Fragrance	x	6	3
Caryophylla-4(12),8(13)-dien-5.beta.-ol	019431-80-2	C15H24O	220.4	Flavoring agents	x	5	1
Perhydrofarnesyl Acetone/Hexahydrofarnesyl Acetone	000502-69-2	C18H36O	268.5	Flavor/Fragrance	x	5	x
Humulene oxide II	019888-34-7	C15H24O	220.4	Terpene	x	5	1
Geraniol	000106-24-1	C10H18O	154.3	Terpene	x	4	x
Rotundifolone/Piperitenone oxide	003564-96-3	C10H14O2	166.2	Natural extract??	x	4	2
Trans-Calamenene	073209-42-4	C15H22	202.3	Terpene	x	4	x
Beta-selinene	017066-67-0	C15H24	204.4	Terpene	x	3	3
Camphene	000079-92-5	C10H16	136.2	Terpene/Flavor/Fragrance	x	3	5
Methyl heptenone	000110-93-0	C8H14O	126.2	Flavor/Fragrance	x	3	x
p-Mentha-1,5,8-triene	021195-59-5	C10H14	134.2	Terpene	x	3	1
Alpha-Bisabolene/(E)-Alpha-Bisabolene	025532-79-0	C15H24	204.4	Flavor/Fragrance	x	2	x
Alpha-Guaiene	003691-12-1	C15H24	204.4	Terpene/Flavor/Fragrance	x	2	2
Calarene/(+)-Calarene	017334-55-3	C15H24	204.4	Terpene	x	2	x
Bicyclo[7.2.0]undec-3-ene, 4,11,11-trimethyl-8-methylene-	889360-49-0	C15H24	204.4	Terpene	x	2	2
Cherry propanol	001197-01-9	C10H14O	150.2	Flavor/Fragrance	x	2	2
Cis-Beta-Farnesene	028973-97-9	C15H24	204.4	Terpene	x	2	1
Bicyclo[7.2.0]undecane, 10,10-dimethyl-2,6-bis(methylene)-	357414-37-0	C15H24	204.4	Terpene	x	x	6
O-Cymene	000527-84-4	C10H14	134.2	Terpene/Flavor/Fragrance	x	x	4
Eudesma-4,6-diene	028624-28-4	C15H24	204.4	Terpene	x	2	x
Gamma-Terpinene	000099-85-4	C10H16	136.2	Terpene/Flavor/Fragrance	x	2	3
Gamma-Terpineol	000586-81-2	C10H18O	154.3	Terpene	x	2	1
Humulenol-II	019888-00-7	C15H24O	220.4	Terpene	x	2	1
Isocaryophyllene	000118-65-0	C15H24	204.4	Terpene	x	2	x
Tricyclene	000508-32-7	C10H16	136.2	Flavor/Fragrance	x	2	1
Ylangene	014912-44-8	C15H24	204.4	Terpene	x	2	2
1,3,8-p-Menthatriene	018368-95-1	C10H14	134.2	Flavoring	x	1	3
1,8-menthadien-4-ol	997077-81-3	C10H16O	152.2	Terpene??	x	1	x
3-Thujene	002867-05-2	C10H16	136.2	Terpene	x	1	x
6,9-Guaiadene	036577-33-0	C15H24	204.4	Flavor/Fragrance	x	1	x
Alpha-Curcumene	000644-30-4	C15H22	202.3	Terpene	x	1	x
Alpha-Himachalene	003853-83-6	C15H24	204.4	Terpene	x	1	x
Alpha-Panasinsen/(-)-Alpha-Panasinsen	056633-28-4	C15H24	204.4	Terpene	x	1	x
Alpha-Terpinene	000099-86-5	C10H16	136.2	Terpene/Flavor/Fragrance	x	1	1
aR-Himachalene	019419-67-1	C15H22	202.3	Terpene	x	1	1
Benzophenone	000119-61-9	C13H10O	182.2	Flavor/Fragrance	x	1	x
Beta-Fenchol/(-)-Beta-Fenchol	000470-08-6	C10H18O	154.3	Terpene	x	1	x
Myrtenol/(-)-Myrtenol	019894-97-4	C10H16O	152.2	Terpene/Flavor/Fragrance	x	1	x
Beta-Patchoulene	000514-51-2	C15H24	204.4	Fragrance/Perfuming	x	1	1
Beta-Vetivenene	027840-40-0	C15H24	204.4	Terpene	x	1	x
Cubenene	029837-12-5	C15H24	204.4	Terpene	x	1	1
Caryophyllene-(I1)	136296-37-2	C15H24	204.4	Terpene	x	1	x
Caryophyllenyl alcohol	913176-41-7	C14H24O	222.4	Flavor/Fragrance	x	1	x
Cis-8-Methylbicyclo[4.3.0]non-7-ene	057497-08-2	C10H16	136.2	Terpene	x	1	x
Clovanediol	002649-64-1	C15H26O2	238.4	Terpene	x	1	x
Delta-Cadinene	000483-76-1	C15H24	204.4	Terpene	x	1	3
Dihydrocarvyl acetate	020777-49-5	C12H20O2	196.3	Flavor/Fragrance	x	1	1
Eugenol	000097-53-0	C10H12O2	164.2	Cosmetic/Flavor/Fragrance	x	1	1
Gamma-Gurjunene	022567-17-5	C15H24	204.4	Terpene	x	1	x
Gamma-Maalinene	020071-49-2	C15H24	204.4	Terpene	x	1	1
Geranyl Acetone	003796-70-1	C13H22O	194.3	Flavor/Fragrance	x	1	x
Humulene epoxide I	019888-33-6	C15H24O	220.4	Terpene	x	1	1
Isobornyl Acrylate	005888-33-5	C13H20O2	208.3	Film forming	x	1	2
Lavandulyl propionate	059550-34-4	C13H22O2	210.3	Terpene	x	1	x
Menthyl acetate	000089-48-5/002623-23-6	C12H22O2	198.3	Flavor/Fragrance	x	1	1
Methyl salicylate	000119-36-8	C8H8O3	152.1	Terpene/Flavor/Fragrance	x	1	1
Olivetol	000500-66-3	C11H16O2	180.2	Natural extract	x	1	x
Phytol acetate	076337-16-1/010236-16-5	C22H42O2	338.6	Flavor/Fragrance	x	1	x
Pseudolimonene	000499-97-8	C10H16	136.2	Terpene	x	1	1
Schyobunol	035727-45-8	C15H26O	222.4	Terpene	x	1	x
Selin-6-en-4.alpha.-ol	118173-08-3	C15H26O	222.4	Terpene	x	1	x
Tetrahydrogeranyl Acetone	001604-34-8	C13H26O	198.3	Flavor/Fragrance	x	1	x
Trans-Farnesol	000106-28-5	C15H26O	222.4	Terpene/Flavor/Fragrance	x	1	x
Zonarene	041929-05-9	C15H24	204.4	Terpene	x	1	x
O-Cymene	000527-84-4	C10H14	134.2	Terpene/Flavor/Fragrance	x	x	4
Trans-Beta-Ocimene	003779-61-1	C10H16	136.2	Terpene/Flavor/Fragrance	x	x	2
Beta-Bisabolene	000495-61-4	C15H24	204.4	Flavor/Fragrance	x	x	2
Cis-2,6-Dimethyl-2,6-octadiene	002492-22-0	C10H18	138.3	Terpene	x	x	2
Beta-Bourbonene/(-)-Beta-Bourbonene	005208-59-3	C15H24	204.4	Flavor/Fragrance	x	x	1
Myrtenol/(-)-Myrtenol	019894-97-4	C10H16O	152.2	Terpene/Flavor/Fragrance	x	x	1
Selina-5,11-diene/(-)-Selina-5,11-diene	997220-96-1	C15H24	204.4	Terpene	x	x	1
Carvomenthene/(+)-Carvomenthene	001195-31-9	C10H18	138.3	Terpene	x	x	1
Menthol/(±)-menthol	001490-04-6	C10H22O	156.3	Terpene/Flavor/Fragrance	x	x	1
4-Terpinenol	000562-74-3	C10H18O	154.3	Flavor/Fragrance	x	x	1
8,9-Dehydrothymol	018612-99-2	C10H12O	148.2	Terpene	x	x	1
Alpha-Bergamotene	017699-05-7	C15H24	204.4	Terpene	x	x	1
Alpha-Elemene	005951-67-7	C15H24	204.4	Terpene	x	x	1
Alpha-Phellandrene	000099-83-2	C10H16	136.2	Terpene/Flavor/Fragrance	x	x	1
Beta-Cubebene	013744-15-5	C15H24	204.4	Flavoring	x	x	1
Beta-Gurjunene	997220-72-3/73464-47-8	C15H24	204.4	Terpene	x	x	1
Cadina-1(6),4-diene	016729-00-3	C15H24	204.4	Terpene	x	x	1
Cyperene	002387-78-2	C15H24	204.4	Terpene	x	x	1
Eremophilene	010219-75-7	C15H24	204.4	Terpene	x	x	1
(R)-Gamma-Cadinene	039029-41-9	C15H24	204.4	Terpene	x	x	1
Isopinocamphone	018358-53-7	C10H16O	152.2	Flavor/Fragrance	x	x	1
Isoterpinolene	000586-63-0	C10H16	136.2	Terpene	x	x	1
Laevo-Bornyl acetate	005655-61-8	C12H20O2	196.3	Flavor/Fragrance	x	x	1
L-Alpha-Terpineol	010482-56-1	C10H18O	154.2	Terpene	x	x	1
Trans-Isolimonene	005113-87-1	C10H16	136.2	Terpene	x	x	1

a Number of samples where each compound was detected.

**TABLE 3:** Cannabinoids and other constituents detected in vape liquid, vapor and aerosol samples.

Compound names	CAS#	Formula	MW	Functions	Liquid^a^	Vapor^a^	Aerosol^a^
Delta9-Tetrahydrocannabinol (delta9-THC)	001972-08-3	C21H30O2	314.5	Cannabinoid	12	10	10
Cannabinol (CBN)	000521-35-7	C21H26O2	310.4	Cannabinoid	12	12	10
Cannabicitran (CBT)	031508-71-1	C21H30O2	314.5	Cannabinoid	12	12	7
Cannabigerol (CBG)	025654-31-3	C21H32O2	316.5	Cannabinoid	11	4	9
Delta9-Tetrahydrocannabivarin (THCV)	031262-37-0	C19H26O2	286.4	Cannabinoid	11	6	8
Cannabichromene (CBC)	020675-51-8	C21H30O2	314.5	Cannabinoid	10	10	10
(6aR,9R)-delta10-THC/(6aR,9S)-delta10-THC	095543-62-7	C21H30O2	314.5	Cannabinoid	9	5	5
Cannabifuran	056154-58-6	C21H26O2	310.4	Cannabinoid	5	x	2
Cannabidiol (CBD)	013956-29-1	C21H30O2	314.5	Cannabinoid	4	x	2
Delta8-Tetrahydrocannabinol (delta8-THC)	005957-75-5	C21H30O2	314.5	Cannabinoid	3	9	4
Cannabicoumaronone	070474-97-4	C21H28O3	328.4	Cannabinoid	2	10	5
Hexahydrocannabinol	006692-85-9	C21H32O2	316.5	Cannabinoid	2	1	x
9(R)-delta6a,10a-THC/9(S)-delta6a,10a-THC	095720-01-7	C21H30O2	314.5	Cannabinoid	2	4	2
Cannabidivarol (CBDV)	024274-48-4	C19H26O2	286.4	Cannabinoid	1	x	x
Delta8-Tetrahydrocannabivarin	031262-38-1	C19H26O2	286.4	Cannabinoid	1	1	x
Exo-THC	027179-28-8	C21H30O2	314.5	Cannabinoid	1	2	x
Cannabivarin (CBV)	033745-21-0	C19H22O2	282.4	Cannabinoid	x	7	1
Olivetol	000500-66-3	C11H16O2	180.2	Natural extract	1	x	x
Linolenic acid, ethyl ester/9,12,15-Octadecatrienoic acid, ethyl ester, (Z,Z,Z)-	001191-41-9	C20H34O2	306.5	Fatty acid	3	2	1
Decanoic acid, methyl ester	000110-42-9	C11H22O2	186.3	Fatty acid	2	x	x
Hexadecanoic acid, ethyl ester	000628-97-7	C18H36O2	284.5	Fatty acid	2	4	x
Linoleic acid	000060-33-3	C18H32O2	280.4	Fatty acid	2	x	x
Linoleic acid, ethyl ester	000544-35-4	C20H36O2	308.5	Fatty acid	2	3	1
Linolenic acid	000463-40-1	C18H30O2	278.4	Fatty acid	2	4	x
Octanoic acid, methyl ester	000111-11-5	C9H18O2	158.2	Fatty acid	2	x	x
Linoleic acid, methyl ester/9,12-Octadecadienoic acid, methyl ester	000112-63-0	C19H34O2	294.5	Fatty acid	1	x	x
Oleic acid, methyl ester	000112-62-9	C19H36O2	296.5	Fatty acid	1	x	x
n-Hexadecanoic acid	000057-10-3	C16H32O2	256.4	Fatty acid	x	3	x
Butanoic acid, ethyl ester	000105-54-4	C6H12O2	116.2	Fatty acid	x	1	x
Oleic acid, ethyl ester	000111-62-6	C20H38O2	310.5	Fatty acid	x	1	x
Hexadecane	000544-76-3	C16H34	226.4	Fatty acid	x	1	x
Hexadecanoic acid, methyl ester	000112-39-0	C17H34O2	270.5	Fatty acid	x	1	x
Linoleic acid	000060-33-3	C18H32O2	280.4	Fatty acid	x	1	x
Decanoic acid	000334-48-5	C10H20O2	172.3	Fatty acid	x	1	x
Octadecanoic acid, ethyl ester	000111-61-5	C20H40O2	312.5	Fatty acid	x	1	x
Octanoic acid	000124-07-2	C8H16O2	144.2	Fatty acid	x	1	x
1-Octadecene	000112-88-9	C18H36	252.5	Fatty acid	x	1	x
1,2-Dioctanoin	001069-87-0	C19H36O5	344.5	MCT	2	x	x
1,3-Dioctanoin	001429-66-9	C19H36O5	344.5	MCT	2	2	x
2-(Decanoyloxy)propane-1,3-diyl dioctanoate	033368-87-5	C29H54O6	498.7	MCT	2	2	2
2-(Octanoyloxy)propane-1,3-diyl bis(decanoate)	033368-86-4	C31H58O6	526.8	MCT	2	2	2
Decanoic acid, 1,2,3-propanetriyl ester	000621-71-6	C33H62O6	554.8	MCT	2	x	x
Decanoic acid, 2-hydroxy-3-[(1-oxooctyl)oxy]propyl ester	093980-84-8	C21H40O5	372.5	MCT	2	x	x
Glycerol tricaprylate	000538-23-8	C27H50O6	470.7	MCT	2	2	2
Glycerol 1,2-diacetate	000102-62-5	C7H12O5	176.2	MCT Derivative	x	1	1
Decaethylene glycol	005579-66-8	C20H42O11	458.5	Solvent	3	x	x
Undecaethylene glycol	006809-70-7	C22H46O12	502.6	Solvent	3	x	3
Pentaethylene glycol	004792-15-8	C10H22O6	238.3	Solvent	2	3	2
Octaethylene glycol	005117-19-1	C16H34O9	370.4	Solvent	2	1	3
Heptaethylene glycol	005617-32-3	C14H30O8	326.4	Solvent	1	x	1
Hexaethylene glycol	002615-15-8	C12H26O7	282.3	Solvent	1	3	1
Nonaethylene glycol	003386-18-3	C18H38O10	141.5	Solvent	1	x	2
Tetraethylene glycol	000112-60-7	C8H18O5	194.2	Solvent	1	1	x
Triethylene glycol	000112-27-6	C6H14O4	150.2	Solvent	x	2	x
Propylene glycol	000057-55-6	C3H8O2	76.1	Solvent	x	x	1
Vitamin E	000059-02-9	C29H50O2	430.7	Food additive	6	x	x
Vitamin E acetate	000058-95-7	C31H52O3	472.8	Food additive	3	3	2

a Number of samples where each compound was detected.

**FIGURE 1:**
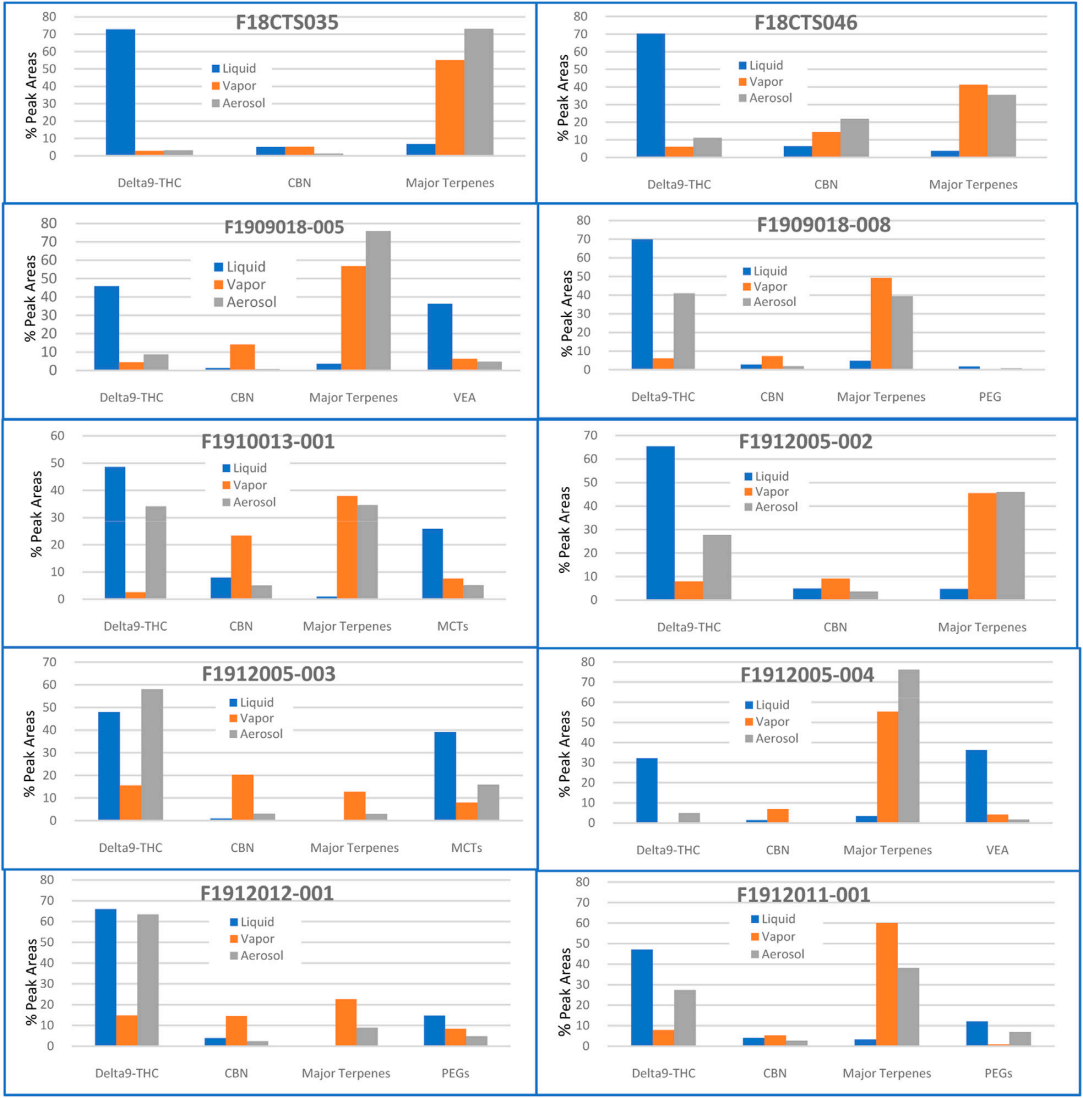
Major terpenes, Delta9-THC, and major additives in vape oil liquid, vapor and aerosol of each vape oil cartridge sample. % Peak Area, area percentage of each peak or compound found in instrument analysis. It roughly represents the composition or amounts present in the sample.

Vape oil liquid samples consisted of small amounts of terpenes from below 1–7% based on the peak areas of the total compounds found in one sample (Figure 1). The most common terpenes and natural extracts found were Caryophyllene (12 samples), Alpha-Bisabolol (11 samples), Linalool (10 samples), Alpha-Humulene (9 samples), Caryophyllene oxide (8 samples), D-Limonene (8 samples), Phytol (8 samples), Fenchol (6 samples), Nerolidol (6 samples), Selina-3,7(11)-diene (6 samples), Squalene (6 samples), Vitamin E (6 samples), Beta-Myrcene (5 samples), and Gamma-Selinene (5 samples). Among these commonly found terpenes, Caryophyllene, D-Limonene, Alpha-Humulene were found at higher percentage compared to other terpenes. These major terpenes we found are consistent with the ones described in USP from cannabis plants (Sarma et al., 2020). In general, typical Cannabis plants can have as many as 140 different terpenes (containing carbon and hydrogen) and terpenoids (containing carbon, hydrogen and oxygen) including monoterpenoids (C10), sesquiterpenoids (C15), diterpenoids (C20), and triterpenoids (C30) (Brenneisen and ElSohly, 2007). In this study we found around 60 various terpenes, terpenoids, flavor and fragrance agents in the twelve tested samples. Although most of these 60 terpenes maybe natural substances carried over through extraction process from cannabis plants, it’s possible that some of the terpenes, especially some flavor and fragrance agents such as Valencene, Menthone, Benzyl Alcohol, D-Carvone, and Triacetin were purposely added into the extracted vape oil to enhance the flavor. Adding flavors into e-liquids for nicotine vaping is a common practice despite the possible health implications (Erythropel et al., 2019) and it is an increasing trend that various terpenes, flavor and fragrance agents are being added into cannabis vape products (Erickson, 2019).

The most common cannabinoids found in this study were Delta9-Tetrahydrocannabinol (Delta9-THC) (12 samples), Cannabinol (CBN) (12 samples), Cannabicitran (CBT) (12 samples), Cannabigerol (CBG) (11 samples), Tetrahydrocannabivarin (THCV) (11 samples), Cannabichromene (CBC) (10 samples), (6aR,9R)-delta10-THC (7samples), and Cannabifuran (5 samples). Delta9-THC was the most dominant cannabinoid in most of the samples with average around 50–60% peak areas of the total compounds found in one sample and with a maximum of 73% peak area (Figure 1). CBN as an oxidation and degradation byproduct of the Delta9-THC was consistently found in all twelve samples ranging from 1 to 20% peak areas of the total compounds. This reveals that the quality of tested vape products has great variations depending on the age of the product, original packing and storage conditions. The amount of CBC, CBG, THCV, CBT and Delta10-THCs also varied from sample to sample.

In two samples (F18CTS035 and F18CTS046) obtained in September and December of 2018, respectively (one year before the EVALI outbreak), the major content was Delta9-THC with greater than 70% peak areas of the total compounds found. CBN as the second major constituent had similar levels of around 5–6% peak areas in both samples and this indicates that vape products may have longer than 12 months of shelf life, especially stored at lower temperatures. Major terpenes and other additives were at much lower levels than cannabinoids with around 7% peak area in one sample and 4% peak area in the second sample (Figure 1).

In the ten samples collected in 2019 during the EVALI outbreak period, Delta9-THC varied significantly ranging from 4.5 to 70% based on the peak areas of the total compounds found in one sample. Five of the samples contained approximately 40–50% of Delta9-THC peak areas in those samples. In the samples with lower levels of cannabinoids, three samples contained more than 30% Vitamin E Acetate (VEA) peak areas of the total compounds with small amount of Vitamin E; two samples contained more than 25% of Medium Chain Glycerides (MCTs) peak areas; three samples contained Polyethylene Glycols (PEGs) with two of them greater than 10% peak areas of the total compounds found; one sample had more than 20% CBN peak area; and one sample had more than 3.5% caryophyllene peak area (Figure 1). In these variety of samples, minor cannabinoids such as Delta8-THC, CBT, (6aR, 9R)-delta10-THC/(6aR,9S)-delta10-THC, 9(R)-delta6a,10a-THC/9(S)-delta6a,10a-THC, Hexahydrocannabinol, Cannabidivarol (CBDV), Delta8-Tetrahydrocannabivarin, and Exo-THC were detected. Usually these minor cannabinoids are seen in the degradation of Delta9-THC or as byproducts of the extraction process in very small amounts (Hudalla, 2020).

Delta10-THCs ((6aR, 9R)-delta10-THC, (6aR,9S)-delta10-THC) have no pharmacological effects and 9(R)-delta6a,10a-THC and 9(S)-delta6a,10a-THC have very low or very limited psychoactivity. These byproducts are not well studied for their efficacy and toxicity and therefore, the long-term health effects in cannabis products are unknown to consumers (Hudalla, 2020; Williams, 2020). Delta8-THC exists in small amounts in cannabis plants (<1%). The identified Delta8-THC isomers may have resulted from raw cannabis plant material from the extraction or post extraction processing using ethanol, hydrocarbons, or CO_2_ to extract/purifying cannabis oil, and remove waxes and chlorophyll (Wilhelm; Hudalla, 2020). Delta8-THC has become increasingly popular and drawn attention in recent days when CBD became nationally legalized. CBD can be easily converted to Delta 8-THC with addition of catalysts (e.g., p-toluenesulfonic acid) in a solvent mixture (Barrie Webster and LeonardSarna, 2004). However, the conversion process is unpredictable in producing other byproducts such as other minor cannabinoids that are not found in natural cannabis plants. Delta 8-THC is not currently covered under the current California cannabis regulations and it has almost two thirds of the psychoactivity compared to Delta9-THC (Inverse.com, 2020). In one sample (F1912013-004), in addition to VEA, more than 30% Delta8-THC and 10% 9(S)-delta6a,10a-THC peak areas were detected, indicating likely adulteration with the synthetic form of Delta8-THC. In these ten vape cartridges, the CBN ranged from 1–20% and most of the samples had 3–5% peak areas. The sample with 20% CBN peak area was half full in an unused cartridge and had sign of drying. This was likely due to a poor sample seal or storage problem.

Vitamin E and VEA were found in six and three of the samples, respectively. Vitamin E, also known as tocopherols, is well known for its antioxidant properties. The most active form of alpha-tocopherol is commonly found in plant material, especially in plants with high oil content and hemp seed (Callaway, 2004). It is essential for plant development and help to provide the major antioxidant function for free radical damages (Muñoz and Munné-Bosch, 2019). Therefore, it is possible to have small amount of Vitamin E in cannabis plant that is coextracted and carried into the vape oil product. However, according to Brenneisen et al., Vitamin K was the only Vitamin found in cannabis plants (Brenneisen and ElSohly, 2007). Large amount of VEA were found in three cartridge samples. VEA is also called α-tocopheryl acetate and is a synthetic form of Vitamin E [EFSA, 2016]. It has a similar appearance to cannabis vape oil. It is commonly added to THC vaping liquids to dissolve/dilute or thicken them as a cutting agent to cut down the cost. Recent studies by the Centers for Disease Control and Prevention (CDC), the US Food and Drug Administration (USFDA), state investigators and research institutes have concluded that VEA is strongly associated with EVALI (Chand et al., 2019; Blount et al., 2020; Matsumoto et al., 2020; Muthumalage et al., 2020). After the EVALI outbreak, VEA is currently banned from cannabis products in many states (Boudi et al., 2019; Gibbons, 2021). In the samples containing large amounts of VEA, Vitamin E was also found and at higher amount than the other three samples with Vitamin E alone. They are probably the byproduct of VEA as VEA is sensitive to hydrolysis and breaks down to free Vitamin E and acetic acid [EFSA, 2016].

Medium-chain triglycerides (MCTs) are getting more attention in recent years for their health benefits as quick energy sources which are less likely to be stored as fat. They are also used as the supplement among athletes and bodybuilders as well as to aid weight loss (Mary Jane Brown, 2020). Triglyceride is simply the technical term for fat and has two main purposes either the body will burn it for energy or store as body fat. MCTs contain two or three fatty acids that have a chain length of 6–12 carbon atoms and they include caproic acid or hexanoic acid (C6), caprylic acid or octanoic acid (C8), capric acid or decanoic acid (C10), and lauric acid or dodecanoic acid (C12). Food sources rich for commercial extraction of MCTs include palm kernel oil and coconut oil (Mary Jane Brown, 2020). Like VEA, MCTs are added to THC e-liquid as dilute or thickening agent based on their appearance and claimed health benefits, especially in counterfeit products (Chand et al., 2019; Muthumalage et al., 2020). MCTs are generally regarded safe by the FDA as food additives under certain limitations (USFDA, 1938a). However, little is known about how they affect the respiratory tract and its local immune-inflammatory functions when used in vape products. MCTs were also found in bronchoalveolar lavage fluid in EVALI patients from CDC’s study (Blount et al., 2019; Blount et al., 2020). In the current study, we found two samples containing around 25 and 39% MCT peak areas with the major compounds as 2-(Decanoyloxy)propane-1,3-diyl dioctanoate, 2-(Octanoyloxy)propane-1,3-diyl bis(decanoate), and Glycerol Tricaprylate.

Polyethylene Glycols (PEGs) were also found in three vape cartridge samples and they contained 2–15% total peak areas including Tetraethylene Glycol, Pentaethylene Glycol, Hexaethylene Glycol, Heptaethylene glycol, Nonaethylene Glycol, Octaethylene Glycol, Decaethylene Glycol, and Undecaethylene Glycol. Propylene glycol (PG) and vegetable glycerin (VG) are two of the primary solvents heavily used in nicotine e-liquid as the thinning agents. PEGs have also been found in vape products (Traboulsi et al., 2020). PEG 400 is a low molecular weight grade of PEG that is widely used in cosmetics and pharmaceutical formulations as a solvent/lubricant due to its low oral and dermal toxicity. Even though they are safe as food additives (USFDA, 1938b), studies have shown that during vaping, PG and PEG 400 produced high levels of toxic compounds-acetaldehyde and formaldehyde when heated to 230°C. In addition, PEG 400 produced significantly higher levels of acetaldehyde and formaldehyde than PG, MCT, and VG (Troutt and DiDonato, 2017). Samples containing PEGs were also found in EVALI patients (USFDA, 2020c).

In these vape oil samples, we also found a set of fatty acids, mainly unsaturated fatty acids including linoleic acid, linolenic acid, oleic acid, and their methyl ester or ethyl ester derivatives (Table 3). These acids are commonly found in cannabis, and are especially rich in cannabis seeds (Callaway, 2004; Brenneisen and ElSohly, 2007). They have some terpene functions and offer flavors such as green, fruity, waxy, citrus, aldehydic soapy, creamy, and coconut (The Good Scents Company I). Other compounds we found were Benzyl Alcohol (1 sample) and Butylated Hydroxytoluene (1 sample) used as preservatives; and Triacetin (1 samples) reported to function as a cosmetic biocide, plasticizer, and solvent in cosmetic formulations. Finally, a small portion of the compounds in each sample could not be identified using Wiley11/NIST 2017 Mass Spectral libraries and Cayman library.

### Headspace GC-MS Screen for Cannabis Vape Oil Vapor

**FIGURE 2:**
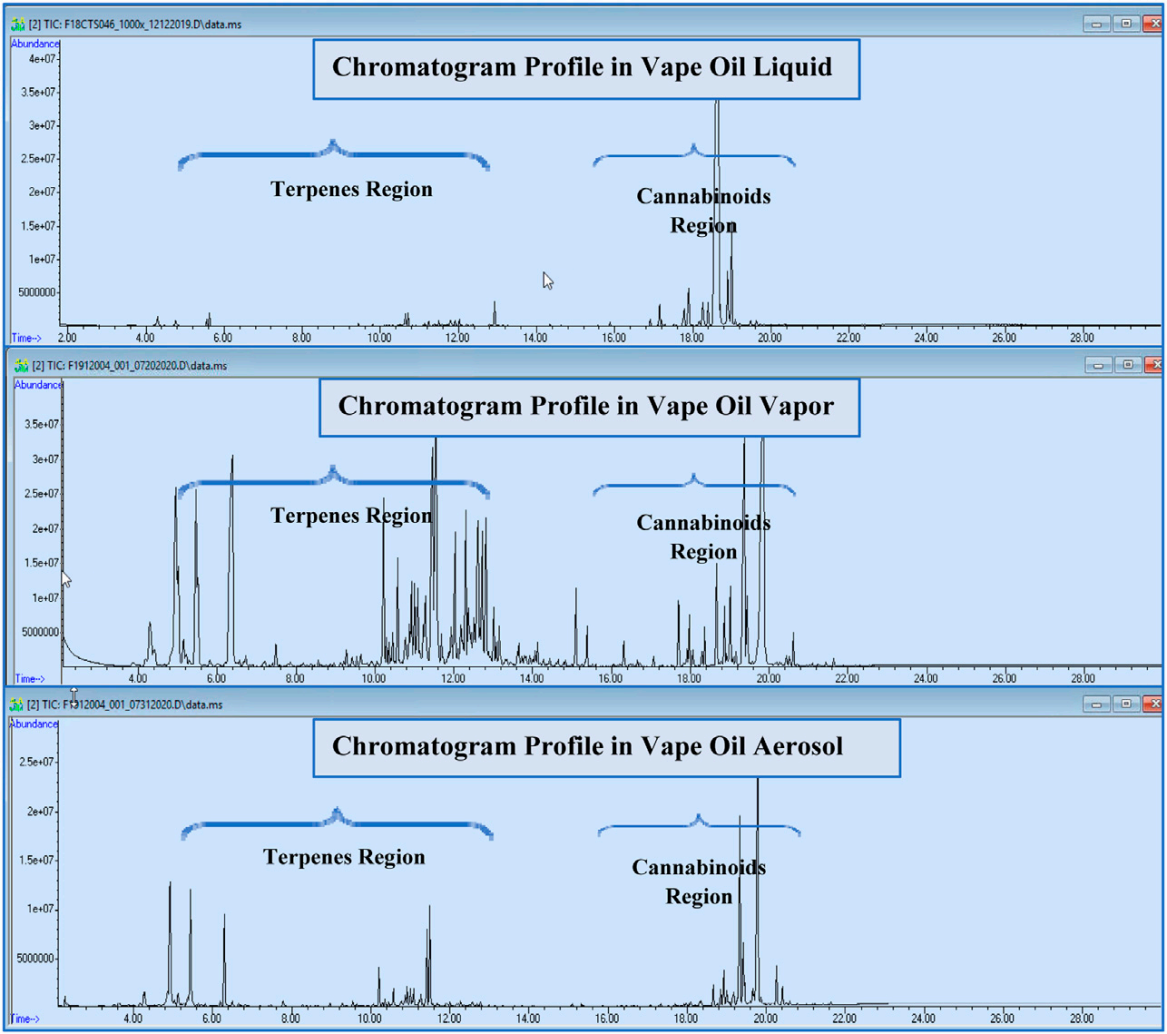
Total ion chromatograms of liquid, vapor, and aerosol phases in one of tested samples.

Twelve cannabis vape oil samples were heated to 200°C to simulate how users consume vape oil. We found that many more terpenes were generated, and the major terpenes were at much higher concentrations in the vapor. The cannabinoid levels were much lower in vapor content compared to the liquid vape oil (Figure 2). In general, terpenes have smaller molecular weight and lower boiling point than cannabinoids. Therefore, heating increased the terpene compositions in headspace vials, resulting in more terpene types and higher amount observed in vape oil vapor samples.

The most common terpenes and natural extracts found were Caryophyllene (12 samples), Alpha-Humulene (12 samples), Alpha-Bisabolol (11 samples), Eudesma-3,7(11)-diene/Selina-3,7(11)-diene (11 samples), Linalool (10 samples), 2-Pinene (9 samples), D-Limonene (9 samples), Phytol (9 samples), Beta-Pinene (2(10)-Pinene) (8 samples), Caryophyllene Oxide (7 samples), Copaene (7 samples), Fenchol (7 samples), Gamma-Selinene (7 samples), Nerolidol (7 samples), 3-Methylcyclopentyl acetate (6 samples), Epi-γ-Eudesmol (6 samples), Piperitenone (6 samples), Terpinolene (6 samples), Alpha-Selinene (5 samples), Beta-Myrcene (5 samples), Caryophylla-4(12),8(13)-dien-5.beta.-ol (5 samples), Endo-Borneol (5 samples), Humulene oxide II (5 samples), Neophytadiene (5 samples). Among these commonly found terpenes, Caryophyllene, D-Limonene, Alpha-Humulene, Linalool, and Terpinolene were dominant. More than 100 terpenes and related compounds were released in vapor samples and they are shown in Tables 2. After heating, total terpenes can comprise of more than 60% peak areas of the total compounds and some major terpenes such as caryophyllene or 2-Pinene can have more than 20% peak area alone (Figure 1).

The most common cannabinoids found were CBT (12 samples), CBN (12 samples), Delta9-THC (10 samples), CBC (10 samples), Cannabicoumaronone (10 samples), Delta8-THC (9 samples), Cannabivarin (CBV) (7 samples), THCV (6 samples), (6aR,9R)-delta10-THC or (6aR,9S)-delta10-THC (5 samples) (Table 3). Delta9-THC dropped significantly to below 15% peak areas in most of the vapor samples (Figure 1). We found minor cannabinoids such as Delta8-THC, Cannabicoumaronone, CBV, and 9(S)-delta6a,10a-THC present in more samples, and CBG, and THCV in fewer samples compared to vape oil liquid. This minor cannabinoid profile change is probably due to the heating process and a study has shown that CBV is an oxidized product of THCV likely due to heating (Bailey and Gagné, 1975). In most of the samples, CBN as a degradation byproduct of Delta9-THC became more dominant than Delta9-THC after heating. This can be beneficial effect as CBN is non-psychoactive with some therapeutic benefit/potential to treat disease. CBN acts as a sedative, anticonvulsant in animal and human studies, and has demonstrated significant properties related to anti-inflammatory and antibiotic activities (Brenneisen and ElSohly, 2007; EthanRusso, 2017).

The potential toxins or additives including VEA, PEGs and MCTs found in liquid injection were also found in vapor samples but at much lower levels (Figure 1). This is likely due to their higher molecular weight and higher boiling points compared to certain terpenes and cannabinoids. VEA and MCTs dropped to below 7 and 9% peak areas in vapors compared to over 30 and 25% peak areas in vape liquid samples, respectively. PEG levels also dropped significantly. Even with smaller amounts released, these additives may pose toxic effects in EVALI patients addressed in previous section. We also found a similar set of fatty acids and they had similar behaviors as terpenes producing higher levels in vapor samples after heating. Vitamin E was not observed in vapor samples as it is unstable at high temperatures and may decay or break down to other unidentifiable compounds (Kuppithayanant, 2014).

### SPME GC-MS Screen for Cannabis Vape Oil Aerosol

Ten out of twelve planned vape oil cartridges were able to be tested for constituents in vape oil aerosol samples. The total aerosol amounts generate from five puffs ranged from 15 to 31 mg. We noticed that some cartridges were much easier to light and generate aerosol while some generated aerosols very slowly. We found that easy lit and aerosolized cartridges typically had lower vape oil viscosities. Two vape cartridges were unable to generate aerosols at all using the same vape device, indicating their poor quality and short shelf life. We also noticed that different puffs generated from the same cartridge can be very different for the amounts of terpenes and cannabinoids released. The lower amounts of the vape oil used to generate aerosols, the higher amounts of the terpenes and the lower amounts of cannabinoids were released, and vice versa. In general, more terpenes were released than cannabinoids in aerosol samples, similar to vapor samples (Figure 2).

In tested aerosol samples, the most common terpenes found were Alpha-Humulene (10 samples), Caryophyllene (10 samples), D-Limonene (10 samples), Eudesma-3,7(11)-diene or Selina-3,7(11)-diene (10 samples), Terpinolene (10 samples), 2-Pinene (9 samples), Beta-Pinene (9 samples), Fenchol (9 samples), Linalool (9 samples), Alpha-Bisabolol (8 samples), Beta-Myrcene (8 samples), Copaene (8 samples), Caryophyllene oxide (6 samples), Bicyclo[7.2.0]undecane, 10,10-dimethyl-2,6-bis(methylene)- (6 samples). Alpha-Selinene/(+)-Alpha-Selinene (5 samples), Camphene (5 samples), Delta-Guaiene (5 samples), Gamma-Selinene (5 samples). Among the most commonly found terpenes, Caryophyllene, D-Limonene, Alpha-Humulene, Linalool, and Terpinolene, and 2-Pinene were the most abundant. More than 100 terpenes and natural extracts were generated through vaping and they are listed in Tables 2. After heating, the major terpenes can have more than 75% of total peak area, more than those of in vapor samples. Some samples contained lower levels of terpenes compared to vapors (Figure 1).

The most common cannabinoids found were Delta9-Tetrahydrocannabinol (Delta9-THC) (10 samples), Cannabinol (CBN) (10 samples), Cannabichromene (CBC) (10 samples), Cannabigerol (CBG) (9 samples), Delta9-Tetrahydrocannabivarin (THCV) (8 samples), Cannabicitran (CBT) (7 samples), (6aR,9R)-delta10-THC or (6aR,9S)-delta10-THC (5samples), and Cannabicoumaronone (5 samples) (Table 3). Similar to the cannabis vapor, we found much higher amounts of terpenes and lower amounts of cannabinoids than vape oil liquid. However, Delta9-THC was at higher levels compared to the vapor and it was still the most dominant cannabinoid in six of the 10 aerosol samples ranging from 27 to 63% peak areas. This indicates that the vape pen used may generate temperatures higher than 200°C. Studies have shown that lower vape temperatures usually provide more terpene flavors, and higher vape temperatures give stronger psychoactive effects (more Delta9-THC content) (vaping360.com, 2020; zamnesia.com, 2020). This is also confirmed from our experiment by heating vape oil in headspace vials at 150°C and 200°C. We observed higher amounts of terpenes and lower amounts of cannabinoids in 150°C vapor samples. Both aerosol and vapor samples produced more terpene types than vape liquid samples, but aerosol produced less terpene types compared to vapor due to higher heating temperatures.

In aerosols, we also found minor cannabinoids such as Delta8-Tetrahydrocannabinol, (6aR,9R)-delta10-THC, (6aR,9S)-delta10-THC, 9(S)-delta6a,10a-THC, and Cannabifuran. This indicates that at the vaping temperature used, minor cannabinoids may be formed from major cannabinoid such as Delta9-THC. The potential toxins or additives including VEA, PEGs and MCTs found in vape liquid and vapor were also found in aerosol samples. Their levels were similar as vapor (Figure 1). Interestingly, Vitamin E was not found and only a few fatty acids were found in aerosol samples probably due to their decaying at high temperatures. The possible breakdown product of toxic gas ketene from VEA during vaping can be directly attributed to the illness in EVALI patients (Attfield et al., 2020; Strongin, 2020; Wu and O’Shea, 2020). However, ketene was not found in tested vapor or aerosol samples.

Some specific terpenes such as Bicyclo[7.2.0]undec-3-ene, 4,11,11-trimethyl-8-methylene-; Bicyclo[7.2.0]undec-3-ene, 4,11,11-trimethyl-8-methylene-; 1,3,8-p-Menthatriene; and Isobornyl Acrylate, and some flavor additives such as Cherry propanol, Citronellol; and Lavandulyl Propionate were observed in both vapor and aerosol samples. More isomers (e.g., alpha, beta, gamma) of some terpenes were found in vapor and aerosols likely due to heat transformation. Some compounds were degradation or derivative products from major terpenes after heating, for example Humulene epoxide I, Humulene oxide II, and Humulenol-II were likely produced from Alpha-Humulene. Caryophylla-4(12),8(13)-dien-5.beta.-ol, Caryophyllene oxide, Caryophyllene-(I1), Caryophyllenyl alcohol, Caryophylla-4(12),8(13)-dien-5.beta.-ol, Isocaryophyllene were likely generated from Caryophyllene. Eudesma-3,7(11)-diene, Eudesma-4(14),11-diene, and Eudesma-4,6-diene were likely generated from Eudesma.

### Strength and Limitations

To our knowledge, this was the first study to provide a comprehensive list of the terpenes, cannabinoids, and additives found in vape oil samples, especially in heated vapor and aerosols. This small study compared the major constituents and potential toxic additives such as VEA, PEG, MCTs in vape cartridges before and after EVALI outbreak and in three different sample forms (liquid, vapor and aerosol). The data generated can aide to assess the types and amounts of the constituents inhaled through vaping by consumers. In this study, only 12 vape oil cartridges were tested. A larger number of samples should be investigated to confirm the current findings. The aerosol experiment was designed to simulate vaping, but it was not identical to vaping by users. The tested conditions used that may differ are: 1) vacuum flow used to generate aerosol can be different from inhaling by a person’s breath; 2) SPME fiber used can only absorb limited amount of volatile and semivolatile compounds; 3) exact vape temperature cannot be measured and it can be different as used by consumers; 4) the same vape device with the same voltage was used which may be different from consumers that use various vape devices with different voltage settings. Finally, various constituents and additives were not accurately quantified in this study.

## Conclusions and Future Studies

In the current study, we have detected over 100 terpenes and natural extracts, 19 cannabinoids including some minor cannabinoids such as cannabicitran (CBT), cannabivarin (CBV), cannabicounaronone, (6aR,9R)-delta10-THC, (6aR,9S)-delta10-THC, 9(S)-delta6a,10a-THC, and Cannabifuran, exo-THC, and Hexahydrocannabinol, as well as other potential toxic additive such as VEA, PEGs, and MCTs in tested vape cartridges. Our study has shown that more terpenes and minor cannabinoids can be produced *via* vaporizing and aerosolizing the vape oil. Delta9-THC and potential toxic additives were found at lower levels in vapor and aerosol samples. Currently, the interactions among high amount of the terpenes released through heating, major and minor cannabinoids, and additives including VEA, MCTs or PEGs, as well as the potential interaction byproducts are not studied. The amounts of cannabinoids inhaled can vary from puff to puff and depend on the quality of the vape oil and devices. Due to the study limitations, we cannot detect other toxins such as ketene that may have direct toxic impact to lung injuries. Even though EVALI outbreak patients have significantly decreased in 2020, they still exist in California during COVID19 pandemic period (Sternlicht, 2020). Therefore, it’s crucial to monitor for potential toxic additives through continuous testing of vape oil products from surveillance and investigations. We are also conducting an experiment to examine cannabis flower aerosol constituents using vaping devices. This study will shed light in discovering potential toxic chemicals formed during dried flower vaping. Furthermore, we have developed a liquid chromatography mass spectrometry (LCMS) toxin screen method to target for nonvolatile constituents and additives to expand the toxin screen capability in our future studies.

## Data Availability

The datasets presented in this article are not readily available because Data can only be released through CDPH Public information officer. Requests to access the datasets should be directed to Gordon.Vrdoljak@cdph.ca.gov.
